# Impact on product quality of high productive GS-CHO cell lines

**DOI:** 10.1186/1753-6561-5-S8-P17

**Published:** 2011-11-22

**Authors:** Angelo Perani, Benjamin Gloria, Dongmao Wang, Roger Murphy, Harjit Khangura Singh, Fiona E Smyth, Andrew M Scott

**Affiliations:** 1 Ludwig Institute for Cancer Research, Melbourne-Austin Branch, Heidelberg, Victoria, 3084, Australia

## Background

The Clinical Program of the Ludwig Institute for Cancer Research (LICR) aims to translate basic laboratory discoveries into early phase clinical trials in cancer patients. A key component of the LICR approach has been to focus on the identification of antibodies selectively targeting antigens preferentially expressed in tumour tissue, and the molecular engineering of chimeric or humanised antibodies to these targets. The development of robust and high producing cell lines is crucial in the development of each antibody construct. The Cell Biology Group at the LICR Melbourne-Austin Branch is responsible for the production of recombinant proteins including monoclonal antibodies from hybridomas or from industrially relevant mammalian cell lines (CHO, NS0, etc). The Cell Line Development activities are driven by the successful combination of industrial relevant cell lines transfected with cancer-relevant DNA targets using the Glutamine-Synthetase system (GS) from Lonza. Here we present two case-studies of high-productive cell lines in terms of product quality and biological activity of the protein produced with these cell lines.

## Material and methods

Cell line development / Production: CHO-K1 SV cells were transfected with the vector coding for monoclonal antibody LICR A within a GS expression vector (Lonza) and maintained in CD-CHO (Invitrogen) + 25μM methionine sulfoximine (MSX) (Sigma). LICR B (mu) murine hybridomas were generated against LICR B antigen and maintained in DMEM + 15% FBS (Invitrogen). Shake flask productivity screens were performed in E250 Erlenmeyer flasks with CD-CHO + 25μM MSX in batch mode for LICR A and LICR B and fed-batch for LICR B. Bioreactor productions: LICR A : 15L stirred-tanks bioreactors (STR) (Applikon) in batch mode + temperature shift in CD-CHO 32μM MSX; LICR B (mu): 15L STR in batch mode + temperature shift in DMEM (Invitrogen) + 15% FBS and LICR B : 7L STR (Applikon) in fed-batch mode + temperature shift in CD-CHO + 32μM MSX. Purification: Supernatants were filtered and purified with Protein A eluted with 100mM glycine (pH 2.5), then adjusted to pH 8.0 with 1M Tris buffer then dialysed into PBS. ELISA assay: An anti-human IgG (γ-chain specific) antibody for coating plates and an anti-human IgG (γ-chain specific) alkaline phosphatase conjugate as secondary antibody. Purified antibody was used to set up a standard curve for calculating antibody concentration. Plates were read at 405 nm.

SDS PAGE: Antibody (5μg) in a final volume of 20μl was loaded under both reducing and non-reducing conditions on NuPage, 4-12% Bis-Tris gels (Invitrogen) along with 20μl of molecular weight standards. Protein bands were detected with Coomassie blue staining. Biosensor analyses were conducted on a BIAcore 2000 instrument (GE). The epitope for the recombinant antibody was immobilized in a Ni-NTA chip and a blank control channel was for correction of refractive index effects. Sample at increasing concentrations were flowed over the chip surface and then washed out. The chip was regenerated with 0.1M EGTA between each analysis. FACS analysis was performed with a FACS Canto II cytometer (Becton Dickinson), Cell line expressing antigen A at 5x10^5^cells/sample; Negative control: isotype IgG (10μg /mL); Positive control: Purified LICR A (10μg/mL); Secondary antibody PE conjugated (1:1000) (Sigma); Data analysis using Gatelogic v 3.08 (Inivai).

## Results – discussion

LICR A: Production of the LICR A antibody increased from 265 mg/L in shake-flask production to 721 mg/L in 15L bioreactor (Results not shown). The FACS analysis of LICR A antibody with cells expressing the A antigen shows an increased binding when using purified LICR A antibody from the bioreactor production at 50 μg/mL (Mean Fluorescent Intensity (MFI) for PE from LICR A shake flask: 4591 and MFI PE from LICR A bioreactor: 9396). Physicochemical analysis by SDS-PAGE of the product shows that the heavy and light chains (under reduced conditions) and the banding pattern (under non-reduced conditions) are typical for IgG. On SE-HPLC the main peak (94.7% for shake flask and 91.1% for bioreactor) is eluting at the expected size of an intact IgG.

**Figure 1:**
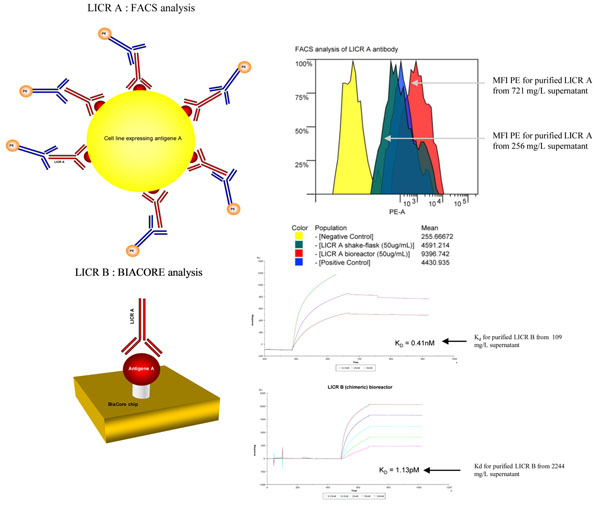
Binding to cells expressing the LICR A antigen analysed by FACS for purified LICR A antibody and Binding to LICR B antigen-coated chips analysed by BIAcore for purified LICR B antibody.

LICR B: Production of the LICR B antibody showed a continued increase from the production in bioreactor of the parental LICR B (mu): 109 mg/L to 537 mg/L in shake-flask batch mode and to 1252 mg/L in shake-flask fed-batch mode (Results not shown). The highest productivity observed by ELISA was observed with the supernatant from the 7L bioreactor in fed-batch mode: 2244 mg/L. The BIAcore comparative analysis of the material purified from the LICR B (mu) bioreactor and LICR B bioreactor fed-batch shows an increased binding (decreased K_d_) : Kd LICR B (mu) bioreactor: 0.41 nM; K_d_ LICR B bioreactor fed-batch: 1.13 pM (Figure 1). Physicochemical analysis by SDS-PAGE of the product shows that the heavy and light chains (under reduced conditions) and the banding pattern (under non-reduced conditions) are typical for IgG. On SE-HPLC the main peak [96.8% for LICR B (mu) and 95.9% for LICR B] is eluting as an intact IgG (Results not shown).

## Conclusion

We have successfully constructed two GS-CHO cell lines producing functional intact antibodies against cancer-related epitopes. The product concentration was substantially increased for both cell lines without having a negative effect of product quality. Analyses of complex glycans present on LICR A and LICR B antibodies are to be performed.

